# Impact of school meals on educational outcomes in Addis Ababa, Ethiopia

**DOI:** 10.1017/S1368980022000799

**Published:** 2022-09

**Authors:** Zelalem Destaw, Eshetu Wencheko, Samuel Kidane, Mulugeta Endale, Yohannes Challa, Melkamu Tiruneh, Meti Tamrat, Hanna Samson, Dilu Shaleka, Mogessie Ashenafi

**Affiliations:** 1 Center for Food Security Studies, College of Development Studies, Addis Ababa University, P.O. Box 1176, Addis Ababa, Ethiopia; 2 Department of Statistics, College of Natural and Computational Sciences, Addis Ababa University, Addis Ababa, Ethiopia; 3 Addis Ababa Health Bureau, Addis Ababa, Ethiopia; 4 Addis Ababa Education Bureau, Addis Ababa, Ethiopia; 5 St. Paul’s Millennium Medical College, Addis Ababa, Ethiopia; 6 College of Development Studies, Addis Ababa University, Addis Ababa, Ethiopia

**Keywords:** Educational performance, Nutrition, School attendance, School feeding, School meals, Social protection

## Abstract

**Objective:**

This study evaluated the impact of the Addis Ababa School Feeding Program (SFP) on educational outcomes.

**Design:**

Single-group repeated measurement/longitudinal study design and multistage stratified sampling design were followed. Effect sizes estimates, repeated measures ANOVA, Chi-square, Generalised Additive Mixed Model and mixed effects negative binomial regression were used. Academic scores, attendance and dropout and height and weight of schoolchildren were collected.

**Setting:**

School Feeding Programs in Addis Ababa, Ethiopia.

**Participants:**

Schoolchildren in primary schools and school directors and teachers in fifteen randomly selected schools for Key Informant Interview (KII).

**Results:**

Anthropometric measurements of 4500 schoolchildren were taken from 50 schools. Academic scores of 3924 schoolchildren from 46 schools, class attendance records of 1584 schoolchildren from 18 schools and annual enrolment records of 50 schools were gathered. School meals achieved a minimum to large scale effects on educational outcomes with effect sizes (*η* ^2^) of academic scores (boys = 0·023, girls = 0·04), enrolment (girls = 0·001, boys = 0·05) and attendance (Cramer’s V = 0·2). The average scores of girls were significantly higher than that of boys (*P* < 0·0001). Height-for-age in all schoolchildren (*P* < 0·01) and BMI-for-age *Z*-scores in adolescent girls of 15–19 years (*P* < 0·0001) never had a significant positive relationship with average scores. Significant relation was observed between nutritional status and attendance (*P* = 0·021). KII showed that SFP created convenient teaching–learning environment and reduced hunger in schools, while boosting enrolment, attendance and academic performance among the schoolchildren.

**Conclusion:**

The Addis Ababa SFP has positively contributed to educational outcomes. Strengthening the program would enhance nutritional outcomes and diminish educational inequalities.

School Feeding Program (SFP) has been a recognised part of the platform for nutritional, health and educational intervention programs such as deworming, micronutrients fortification and supplementation and curricular programmes^(1)^.

Recent studies show different findings about the contribution of SFP with regards to outcomes of energy intake, micronutrient status, enrolment and attendance and academic achievement^(2–5)^. Its positive impact on physical growth, cognitive and academic performance was less conclusive in some countries^(3)^ while substantial helped elsewhere^(2,4–6)^.

In some countries, SFP has increased enrolment in both sexes, and a combination of take-home rations (THR) with on-site feeding sustained enrolment of girls^(7)^. SFP is also believed to pave the way to achieve SDG^(8)^ and to reduce inequalities in education^(9)^.

In sub-Saharan African countries, SFP showed encouraging effect on learning outcomes and small average effect on attendance^(10)^. SFP has made marginal improvement in educational outcomes in countries with fast growing economy^(11)^.

Evidence on the nutritional and educational effects of SFP in Ethiopia is scanty. Yet, since it was established very recently, there is no evidence on the effect of the Addis Ababa SFP on educational outcomes. An earlier study in Sidama Zone, Southern Ethiopia, showed that SFP had no significant impact on school enrolment, attendance and dropout^(12)^; however, later on, reported for the same Zone that SFP encouraged better attendance, academic performance and less dropout^(13)^. This may be associated with the improvement of the SFP through time. In addition, SFP diminished number of absenteeism in *Boricha* district, Southern Ethiopia^(14)^ and in Addis Ababa^(15)^.

Despite those mixed findings, the Addis Ababa SFP was initiated in order to avoid hunger in schools, especially, for the number of those from poor households. As a result, it can be expected that the nutritional status of the schoolchildren would improve, and hence, concentrating in classes reduces absenteeism that might be associated with hunger and health matters due to malnutrition and achieve good educational performance.

The objective of this study was, therefore, to evaluate the impact of the Addis Ababa SFP on educational outcomes of schoolchildren. It examined academic performances, enrolment and attendance rates of primary schoolchildren before and after SFP has been started and explored associations between nutritional status and academic performance of the beneficiary schoolchildren.

## Methods

Mixed research methods of quantitative and qualitative study design were employed. A single group repeated measurement/longitudinal study was designed as an optimal study design due to the fact that the Addis Ababa SFP was universal to schoolchildren attending school. As a result, establishing a control group was not possible.

Subjects of the study were primary schoolchildren involved in the SFP of the Addis Ababa City Administration.

The sample size for the quantitative data was determined based on a single-group repeated measures (longitudinal) study design. Thus, the sample size determination considered within-subject correlation and the effect size of an intervention over time^(16)^. Another important parameter of interest considered was the power of a statistical test, which is the probability to reject a null hypothesis when it is false, i.e. the probability that it will result in the conclusion that the phenomenon-exists, for instance, a positive outcome of school meals on targeted children^(17,18)^.

Therefore, using the above parameters, the sample size for the current single-group repeated measurement study was determined using the formula:

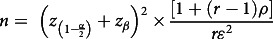

where 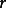 is the number of time points of measurement; 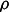 is the assumed correlation of the repeated measures; 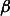 is the desired probability of rejection of null hypothesis and 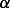 is the desired level of significance.

Since there were no prior estimates of effect sizes and correlations for the proposed study design, we adapted Cohen’s classification of effect sizes of interventions assuming different levels of correlation^(16)^. Accordingly, with a medium effect size of the intervention (0·3), for correlation level of 0·5, at 5 % level of significance, 80 % power, design effect of 1·5 and adjusting for 90 % response rate, a total sample size of ninety targeted schoolchildren, per school, were considered.

Then, a random sample of five schools in each of the ten sub-cities of Addis Ababa was selected that made up a total of fifty schools under consideration for the overall study. Sampling frame, a complete list of schoolchildren, who were involved in the programme at the beginning of this study, was first collected from all the fifty schools. Then fifteen schoolchildren were randomly selected from each first–sixth grades in a school in order to maintain age and sex proportions in the sample. Thus, a grand sum of 4500 schoolchildren involved in the Addis Ababa SFP was targeted.

More information about sample size determination, taking anthropometric measurements, calculation of *Z* scores and estimation of prevalence of malnutrition such as stunting and thinness and overall study design can be found in our previous publication^(19)^.

Data on academic performance, attendance and enrolment and anthropometric measures such as height and weight were collected. Data on average semester scores and attendance (the number of days a schoolchild was absent within a semester) were collected at the end of three consecutive semesters or terms, namely: (i) Semester I – 2011EC (January 2019); (ii) Semester II – 2011 EC (June 2019) and (iii) Semester I – 2012 EC (January 2020). The number of all enrolled students from grade one to grade eight per school was taken for three consecutive academic years, 2018, 2019 and 2020. New academic year in Ethiopia begins in September/October. Anthropometric measurements were measured between June 2019 and January 2020.

Since the Addis Ababa SFP was implemented as of February 2019, the data taken from the first term recordings were considered to define baseline status of educational outcomes. Thus, the second and the third terms represented mid-term and end-term outcomes, respectively, during which schoolchildren had been served school meals in schools.

Descriptive statistics, Chi-square, *t*-tests, repeated measure ANOVA, Generalised Additive Mixed Model and mixed effects negative binomial regression models were used to analyse the data, wherever appropriate in the subtopics presented in the Results section. Height-for-age and BMI-for-age *Z*-scores were used to determine nutritional status and compare the educational outcomes of the schoolchildren against the status of their physical growth gained during June 2019 and January 2020.

Repeated measures ANOVA was used to estimate effect sizes of differences in means^(16,20)^ and Chi-square tests to that of proportions^(21)^. Effect sizes ( 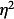 ) of 0·01, 0·06 or 0·14 represent small, medium or large effect, respectively, when repeated ANOVA is used^(16,20)^. Cramer’s V of 0·1–0·2, 0·2–0·4 or 0·4–0·6 refers to weak, moderate or relatively strong association, respectively, when measures of association are used^(21)^.

The qualitative data were collected through Key Informant Interviews (KII) held among school teachers and school directors in March 2021. Interviews were held until a state of data saturation was reached, i.e. until information obtained from the KII sufficient to replicate the study^(22)^ was reached from a total of fifteen randomly selected schools among the fifty target schools selected for this study.

The KII were conducted in Amharic language, using semi-structured interview guides which included the educational benefits of the SFP to the schoolchildren as well as challenges and opportunities.

Each key informant was interviewed separately for about 20–30 min. The researcher began with an introductory interview and probes with follow-up questions to gain more information on the issues raised or any other new information that might be raised by the interviewees. KII were also recorded along with notes taken.

KII recordings and notes were then transcribed in English. The ideas were organised into themes and synthesised. Statements of the respondents that gave emphasis to the issues raised under the themes were quoted, and lessons or conclusions derived out from it were highlighted.

## Results

Anthropometric measurements were achieved for 4500 schoolchildren (46·2 % girls) in 50 schools. Academic scores of 3924 schoolchildren (45·7 % girls) from 46 schools, semester-based class attendance records of 1584 schoolchildren from 18 schools and annual enrolment records of 50 schools were gathered. Since schools were shut down following the COVID-19 incidence, it posed a challenge to access the academic recordings from all of the target schools. As a result, lower sample size was achieved than initially aimed at educational indicators.

### Nutritional status

The prevalence of stunting and thinness during June 2019 and January 2020 has shown a significant difference among boys and girls of all age groups (*P* < 0·01), while the prevalence has generally declined over time. The changes in the sample size per age group in each semester are expected as age increases along with semesters count on (Table 1).

**Table 1:** Prevalence of stunting and thinness by age groups, sex and time

Age (years)	Sex	Stunting (%)			Thinness (%)			*n*	
		June 2019	January 2020	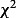	June 2019	January 2020	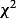	June 2019	January 2020
5–9	Girls	12·2	7·8	6·7**	13·9	12·4	3·5	632	566
	Boys	16·0	10·8	8**	18·9	14·2	10·7**	719	639
10–14	Girls	28·3	22·3	11***	15·5	14·0	5·9	1015	1068
	Boys	30·0	24·7	9**	23·6	21·3	11·9***	1224	1291
15–19	Girls	26·0	20·6	0·5	8·0	3·2	1·5	50	63
	Boys	39·6	33·3	0·5	24·5	24·2	1·6	53	66
All age	Girls	22·2	17·4	12·5***	14·7	13·0	9·6**	1697	1697
	Boys	25·2	20·5	12·3***	21·9	19·1	19·6***	1996	1996
	Both	23·8	19·1	24·6***	18·6	16·3	27·9***	3693	3693

** *P* < 0·05.

*** *P* < 0·01.

*χ* ^2^ is Chi square, tests difference in prevalence between June 2019 and January 2020.

### Academic performance

Average semester scores of boys and girls have increased during each semester between January 2019 and January 2020. ANOVA showed that the mean scores of girls were significantly higher than that of boys at all age groups and during each of the three semesters between 2019 and 2020 (Table 2).

**Table 2:** Mean and standard deviations of scores of schoolchildren by age and sex groups

Semester	Age category (years)	Girls			Boys			ANOVA†, *F*	
		*n*	Mean	sd	*n*	Mean	sd	Sex	Age
January 2019 (Baseline)	5–9	689	72·6	13·2	798	71·5	13·3	45·5***	8·7***
	10–14	1051	72·3	12·5	1277	68·6	12·4		
	15–19	55	73·4	13·8	54	67·5	14·1		
	Total	1795	72·5	12·8	2129	69·7	12·8		
June 2019 (Midline)	5–9	687	73·1	12·8	798	71·6	13·2	45·5***	3·5**
	10–14	1053	72·9	12·5	1277	69·1	12·2		
	15–19	55	74·4	15·4	54	67·8	14·8		
	Total	1795	73·0	12·7	2129	70·0	12·7		
January 2020 (Endline)	5–9	621	74·9	12·4	718	73·5	12·5	52·4***	4·5**
	10–14	1106	74·5	12·6	1344	70·6	12·8		
	15–19	68	77·1	14·9	67	69·0	14·2		
	Total	1795	74·7	12·6	2129	71·5	12·8		

*P < 0·1.

** *P*< 0·05.

*** *P*< 0·01.

† ANOVA test for sex and age differences by semester.

A repeated measures ANOVA showed significant differences in the average scores of the schoolchildren over time (*F*_(2,7846)_ = 108·9, *P* < 0·0001), indicating an increasing trend or improvement in the educational performance of schoolchildren over time. The estimated average effect size ( 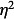 ) was 0·03 (boys = 0·023, girls = 0·04), indicating that the SFP had above a minimum perhaps close to a medium effect on the average academic performance of the schoolchildren. An effect size ( 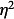 ) of 0·01, 0·06 or 0·14 is considered to be small, medium or large effect, respectively^(16,20)^.

Effect size was minimum for boys of age 10–14 years old ( 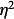 = 0 02) and maximum for girls of age 15–19 years old ( 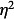 = 0 05) (online Supplementary Table 1).

Generalised Additive Mixed Model also showed that the average scores of the girls were consistently significantly higher than those of boys (*P* < 0·0001). Moreover, it was observed from the model that higher height-for-age *Z*-scores in all schoolchildren (*P* < 0·01) and higher BMI-for-age *Z*-scores in adolescent girls of age 15–19 years (*P* < 0·0001) were significantly related with higher average scores.

**Fig. 1:**
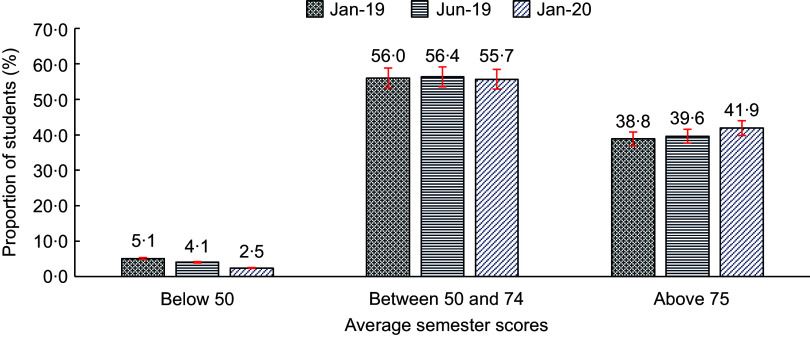
Proportion of schoolchildren by average scores from January 2019 to January 2020

The overall progress in the academic performance is presented in Fig. 1. Among the 3924 schoolchildren followed for their average scores, the proportion of schoolchildren who scored below 50 average points decreased by half during January 2019 and January 2020, while that of those who scored 75 and above increased by about 8 % (3·1 percentage points).

### Enrolment

The total number of boys and girls enrolled each year, the test for the mean size of classes/grade levels each year, the rate of changes from the base year enrolment, September 2018 (2011EC) and the tests for the mean of rates are presented in Table 3. The number of girls enrolled was higher than boys, each year, and *t*-tests confirmed that the average number of boys and girls enrolled were significantly different for each of the 3 years. Enrolment of boys in September 2019 (2012EC) had increased by 2·6 % of that in 2011 and 7·8 % higher number of boys were enrolled in 2020 (2013EC).

**Table 3:** Enrolment and rates of changes over the years by sex

Month/year	Enrolled				Rate of changes			
	Boys	Girls	Both	*t*-value	Boys	Girls	Both	*t*-value
09/2018 (2011EC)	22 545	26 494	49 039	12·0***	Baseline			
09/2019 (2012EC)	23 138	26 688	49 826	11·6***	2·6	0·7	1·6	1·21*
09/2020 (2013EC)	24 309	26 740	51 049	8·7***	7·8	0·9	4·1	4·4***

EC: Ethiopian Calendar.

* *P* < 0·1.

*** *P* < 0·01.

The test for equality of the means of the differences between enrolments in 2019 (2012EC) or 2020 (2013EC) and that of the base year, 2018 (2011) showed that a significantly higher number of boys had registered each year than girls. A similar size of enrolment has been achieved in girls.

**Fig. 2:**
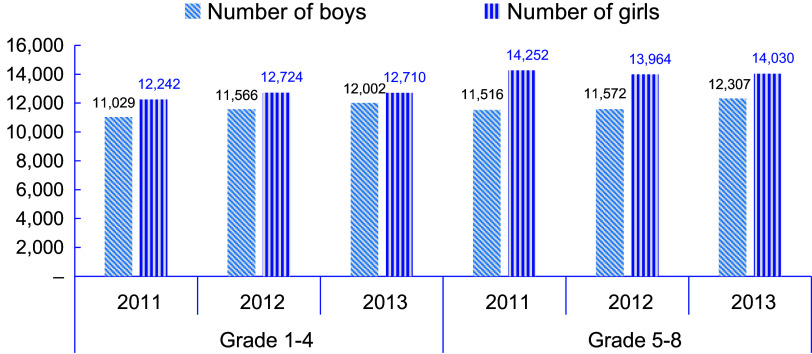
Enrolment by sex from 2011EC (2018) to 2013EC (2020)

The enrolment trend, in number, by grade level and sex groups over the three academic years of 2018, 2019 and 2020 (or 2011, 2012, 2013EC) is presented in Fig. 2. Similar to the results shown in Table 3, the enrolment of all boys and girls in grades one to eight has shown a slight increment, and of these, those in grades five to eight had higher numbers. This highlights that SFP had encouraged adolescent children to come back to school. KII also pointed out that SFP attracted a number of adolescents to school since their parents could send them to school instead of demanding them to work and assist in supporting the family.

Furthermore, a repeated measures ANOVA showed that the number of girls enrolled each year did not change significantly ( 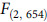 = 0·27; 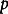 = 0·76; effect size, 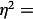 0·001), whereas a statistically significantly higher number of boys had been enrolled at each of the academic years ( 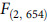 = 18·2; 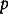 < 0·0001; effect size, 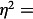 0·05). This indicates that SFP had nearly medium effect among boys’ enrolment while its effect for girls’ enrolment was nil or very little.

### Attendance

Continuous full attendance (with no absenteeism) increased from 72·1 % (baseline) to 77·9 % (2^nd^ assessment) and, finally, to 88·2 % (endline). Majority of schoolchildren were absent from school for less than 5 d. The number of schoolchildren absent for 5 d or more has declined from 46 in 2019 to 12 in 2020 (Table 4).

**Table 4:** Frequency of number of absent days during the three semesters each

Semesters/absentees	January 2019		June 2019		January 2020	
	*n*	%	*n*	%	*n*	%
0	1142	72·1	1234	77·9	1397	88·2
1	178	11·24	125	7·89	56	3·54
2	133	8·4	111	7·01	71	4·48
3	58	3·66	46	2·9	39	2·46
4	32	2·02	22	1·39	9	0·57
5+	41	2·59	46	2·90	12	0·76
Total	1584	100	1584	100	1584	100

For convenient description, the number of absentee days was grouped into ‘No absenteeism’ and ‘Any absence’.

The overall proportion of absenteeism was tabulated for the three consecutive periods/semesters of this study (Table 5). The rate of absenteeism declined from 27·9 % as of January 2019 to 11·9 % by January 2020 (drop off by 16 percentage points, or 57·4 %). Conversely, it implies that the attendance rate raised by 16 percentage points or 57·4 % during the same time of interval. The differences in absenteeism over the periods were significant (*P* < 0·0001) indicating that improvement in class attendance among schoolchildren. Effect size on attendance (Cramer’s V) is 0·2 which corresponds to moderate effect^(21)^ in support of the Addis Ababa SFP had medium effect on attendance.

**Table 5:** Attendance rates and average absent days per student, over time

Attendance	January 2019		June 2019		January 2020		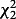
No absenteeism	1142	72·1	1234	77·9	1396	88·1	129·3***
Any absence	442	27·9	350	22·1	188	11·9	
*n*	1584		1584		1584		
Total no. of absent days	1019		901		427		
Mean	0·64		0·57		0·27		

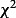
 – Chi square.

Chi-square test showed that statistically significant differences in absenteeism among children of different age groups, regardless of differences in sex each year. However, the attendance rates had improved over time as absenteeism of boys and girls have declined during each semester between January 2019 and January 2020. But absenteeism did not vary significantly with sex (Table 6).

**Table 6:** Association between absenteeism and age groups and sex over three semesters of School Feeding Program period

Semester	Sex	Girls†						Boys†				All ages‡					
	Age group	5–9		10–14		15–19		5–9		10–14		15–19		Girls		Boys	
	Attendance	*n*	%	*n*	%	*n*	%	*n*	%	*n*	%	*n*	%	*n*	%	*n*	%
January 2019	No absenteeism	178	65·9	318	75·2	14	56	194	66	414	76·4	24	80	510	71	632	73
	Any Absence	92	34·1	105	24·8	11	44	100	34	128	23·6	6	20	208	29	234	27
	Total	270	100	423	100	25	100	294	100	542	100	30	100	718	100	866	100
Junee 2019	No absenteeism	189	70·5	340	80	20	80	205	69·7	454	83·8	26	86·7	549	76·5	685	79·1
	Any absence	79	29·5	85	20	5	20	89	30·3	88	16·2	4	13·3	169	23·5	181	20·9
	Total	268	100	425	100	25	100	294	100	542	100	30	100	718	100	866	100
January 2020	No absenteeism	216	86·8	398	90·5	29	93·6	227	84·4	508	89·1	27		641	89·3	756	87·3
	Any absence	33	13·3	42	9·6	2	6·5	42	15·6	62	10·9	7	20·6	77	10·7	110	12·7
	Total	249	100	440	100	31	100	269	100	570	100	34	100	718	100	866	100
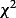	January 2019	9·7***						11·2***				0·74					
	June 2019	8·4**						23·8***				1·58					
	January 2020	2·9						5·7*				1·55					

* *P* < 0·1.

** *P* < 0·05.

*** *P* < 0·01.

† Chi-square (*χ* ^2^) between absenteeism and age group by sex at each semester.

‡ Chi-square (*χ* ^2^) between absenteeism and sex at each semester.

Mixed effects negative binomial regression showed that the estimated effect of a unit changes in height-for-age *Z*-scores resulted in 8% change in the number of class attendances (*P* = 0·021). Thus, better nutritional status might have reduced the frequency of absenteeism, showing that reduction in absenteeism was associated with the Addis Ababa SFP. The KII findings supported this empirical evidence that teachers and school directors noticed dramatic fall in absenteeism.

### Findings from KII

According to the school directors and teachers participated in this study, SFP has reduced absenteeism and no student has been absent from school in connection with the inability to get a lunchpack from home or take breakfast at home. A teacher shared his experience in this as follows:

*Sometimes, schoolchildren did not come to school. We asked their parents. They told us schoolchildren did not go to school because parents were not able to pack lunch for their children. But now parents send their children without any such problem*. (Teacher)

Thus, SFP solved problems that caused absenteeism. In fact, key informants asserted that since the SFP provided both breakfast and lunch every day, schoolchildren rather came to school very early in the morning. This early arrival of the schoolchildren at the schools has now simplified even the teaching–learning process since monitoring and managing late comers and absentees were no more required.

Participants also witnessed that enrolment, attendance, dropout and academic performance of schoolchildren and overall educational activities have improved since the establishment of the SFP, which has resolved multiple problems, such as hunger manifestations at schools and avoiding late coming. According to a school director:

*A five-grader girl, about 16 years old or below, used to be late. I always saw her standing outside the classroom. I asked her homeroom teacher and he said that he was not allowing her to get in because she came to class late. I asked her why she was always late. She showed me her arms putting up her sleeves soiled with dough. I saw that she came to school after baking injera the whole night. She had to finish baking injera before she came to school. She even did not take breakfast. Now it is solved*. (School director).

Thus, the number of latecomers has decreased, schoolchildren attended classes regularly and the teaching–learning process has been smoothly facilitated.

The SFP has created convenient teaching–learning environment. Teachers said that unlike the previous academic environment before the establishment of the SFP, nowadays, teachers could strictly follow-up schoolchildren to attend classes actively and do their homework regularly. Teachers added that they compromised follow-up of the schoolchildren earlier because they knew that schoolchildren from destitute families had to use the time out of school to work or beg along the streets for their lunch or dinner, instead of doing homework and reading at home. Thus, previously, teachers and school directors had difficulty to demand a student, who was under such deprivation, for active class activity and regular homework delivery. A teacher said that:

*I teach Biology. I have seen the difference between teaching well-fed and starved schoolchildren. I was able to read their feelings and pain when they were hungry. We observed that even clever schoolchildren could not participate actively in class activities due to the hunger effect. But now, even the so-called lazy schoolchildren actively participate*. (Teacher).

Another teacher said that:

*The difference is visible before and after SFP was started. Dropout has declined. Teachers used to be too frustrated to give classwork, homework, etc. But now teachers teach without any frustration since they do not see any bad feeling from the schoolchildren’ face as a result of hunger*. (Teacher).

Teachers and school directors also pointed out that the SFP has helped the schoolchildren to improve their academic performance. A school director expressed his experience that:

*I myself have studied the problem with colleagues and found that radical enhancement of some schoolchildren in academic performance with SFP. This showed us that the schoolchildren had the capacity to perform satisfactorily but due to burden of economy and livelihood, their capacity was undermined. Thus, SFP definitely boosted performance of schoolchildren. There were schoolchildren whose parents were at low economy level but academically sound. There were many schoolchildren who had the knowledge and skill, however due to their poor livelihood they dropped out. There were schoolchildren missing from schools at lunch time before SFP. Later on in those days, we saw them along the streets in Merkato begging for money. However, when the SFP reduced their economic burden, they completely stopped disappearing from school at lunch time. They attended all class of the day with full attention and participation*. (School director).

This is indicative that the provision of school meals has a lot of merits and drives smooth delivery of education in schools.

## Discussion

The current finding showed that the Addis Ababa SFP had significantly improved academic outcomes. The program had at least a minimal effect on the average academic performance of the schoolchildren, which is concurrent to findings of other studies. This study has also shown that the Addis Ababa SFP had a positive effect in academic performances of schoolchildren boys of age 10–14 years, but the effect was more noticeable for late adolescent girls of 15–19 years.

Similar effect sizes, measured in terms of Cohen’s f, in academic performance of SFP were reported in Kenya ( 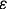 = 0·26) and Senegal ( 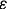 = 0·14) from studies on test scores of schoolchildren served school lunch^(10)^. An effect size (Cohen’s f) of 0·1, 0·25 or 0·4 was considered to be small, medium or large effect, respectively^(16)^.

Overall, a significant increment of enrolment each year has been noted among boys in the 3 years considered in this study. This is in agreement with the education statistics for the year 2019–2020 by the MoE–Ethiopia that the net intake rate at grade one was higher for males than females, where the national target of net intake rate was shown to be met for males^(23)^. Thus, the Addis Ababa SFP that has been going on since February 11, 2019 (*Yekatit* 2011 EC)^(24)^ might have attracted more boys for schooling.

Despite the fact that SFP have potential impacts on education outcomes, the so called low- and middle-income countries focus only on the assessment of its impact on enrolment and attendance^(25)^.

Nevertheless, the findings in this study are comparable to the existing scanty literature about the effect of school meals on academic performance. For instance, better status in height-for-age was linked to academic performance among schoolchildren in *Goba* town, South East Ethiopia^(26)^. In another study in Ethiopia, a significant but small 2·4 percentage points increase in academic performance was observed in SFP beneficiaries^(13)^. In Northwest Ethiopia, low educational performance was significantly associated with stunting, underweight and wasting^(27)^. Conversely, weekend feeding programs improved academic performance in the United States^(28,29)^.

Unlike the finding in this study, the impact of Chile’s SFP had not been shown to increase scores of national mathematics and language tests^(11)^.

Long-term access to school meals influences positive learning achievements. The Indian midday meals is an example that resulted in robust positive effect on learning achievement in India^(30)^.

Similarly, SFP beneficiary children had better performance on national academic assessment tests in Guyana^(31)^, while in Egypt, school meal improved visual memory, auditory vigilance, afternoon attentiveness and working memory^(32)^. In contrast, Argentina recorded only partial improvement in school performance due to the nutritional deficit of the meal^(33)^. In Ghana, SFP improved academic performance and health outcomes,^(34)^ and in the United Kingdom, school meals improved educational outcomes significantly^(35)^. Therefore, consistent with the majority of these studies, the findings in the current study, where academic performance has been at least minimally boosted, would be attributed to an outstanding contribution of SFP of Addis Ababa.

On the other hand, the increase in boys’ enrolment is consistent with that in rural Bangladesh where THR program resulted in an 8% increase in primary school enrolment^(36)^. SFP studies in Ghana consistently reported that an increased enrolment has been linked to school meal provisions^(37)^.

SFP particularly raised girls’ enrolment by 3·2 percentage points in Burkina Faso^(38)^ which was higher than that in our finding (0·9 %). In Uganda, SFP helped girls to timely begin/enroll at first day of schooling^(39)^.

The Chile’s SFP had also shown no evidence in favour of enrolment and attendance^(11)^. The highest earliest baseline enrolment in the education outcomes of Chile and the larger context of Chilean development seem to have dominantly influenced the positive educational outcomes over the SFP^(11)^. However, the researchers further explained that Chile’s SFP significantly influenced educational outcomes when poverty and malnutrition rates were high during which enrolment was also low^(11)^.

In a different study, the overall impact of school meal interventions on education was reported to be strongest where enrolment is low and food insecurity was high^(40)^. An experimental study also revealed that higher attendance has been recorded as a result of SFP among lower-income countries^(41)^. In Mali, SFP motivated child labour reduction, increased enrolment by 10 percentage points and reduced workload and time spent on work among girls^(42)^.

As the findings in this study indicated, the baseline high enrolment rate of girls and lower rate of enrolment of boys might have influenced the significant cumulative difference. While the key message here might be that boys had been deprived of education earlier and might have been forced to dropout or did not enrol at all due to poverty, child labour or hunger. Thus, it can be concluded that the recent progressive rise in the enrolment among boys is associated with the Addis Ababa SFP which avoided the need for child labour and hunger risks among boys. The KII employed in this study confirmed that many schoolchildren used to casually work in the streets or sold small things in local market places to generate income for themselves and their parents as well.

Thus, in sum, it is conclusive that the Addis Ababa SFP contributed to a significant but medium enrolment rate among middle- and late adolescent age boys.

The current findings also showed that the Addis Ababa SFP had medium effect size on school attendance. The finding here varies from that reported by other studies in Ethiopia and elsewhere. In Boricha district, Southern Ethiopia, significantly lower average absentee days were noted among SFP beneficiaries than non-beneficiaries perhaps the mean absent days were larger^(14)^.

On the other hand, evidence shows that school absenteeism was linked to child labour on farms and households to generate income for family food consumption^(43)^ in Malawi. The rise of attendance rate in this study is at least twice as higher as the rate gained among other thirty-two African countries, where school meal provision contributed for 28 % and 22 % rise of girls’ and boys’ school attendances, respectively^(7)^. It is also noted in Burkina Faso that THR increased average school attendance rate for both boys and girls by 8·4 percentage points^(38)^.

The percentage points of attendance gained in this study was higher than that found in Guyana, where 4·3 % increase of attendance was noted as a result of SFP^(31)^. Similarly, in the United Kingdom, school meals contributed for drops in absenteeism by about 0·6 percentage points^(35)^. In addition, in the United Kingdom, school meals were also credited for the decline of authorised absences by 14 %, which may be, they argue, linked to illness and health, as well^(35)^.

Relatively closer to the current findings is that in rural Bangladesh where THR program resulted in a 12 % increase in school attendance^(36)^, and in another study of the same programme, the THR increased school participation by 19 percentage points for boys and 18 percentage points for girls on average^(44)^. In contrast, little improvement in attendance was reported in Ghana^(37)^.

THR and SFP schemes in Uganda achieved large impacts on school attendance and reduced grade repetition^(39)^. Further, in Lao, there was minimal evidence in support of the school feeding schemes increasing enrolment^(45)^.

The discrepancy between the higher impact on attendance in Addis Ababa and lower in other studies may be associated with different reasons. The first reason goes for the difference in the study design. The findings in the other studies were mainly based on cross-sectional comparative settings between beneficiaries and non-beneficiaries. Our study, however, made repeated measurements of a single group. Our findings were limited to the intervention group, however, since the Addis Ababa SFP was universal to establish control group and compare changes in academic performance, attendance and enrolment.

The other reason may be related to socio-economic variations between the countries. For instance, in the United Kingdom, wide difference in the effect size on attendance rate seems plausibly attributed to the larger difference in economic development and initial status of educational outcomes^(35)^. Similarly, in Chile, insignificant effect on educational outcomes was attached to the country’s progressive economic and educational development^(11)^.

In Ethiopia, poverty is among the leading factors responsible for limited school attendance and higher dropout rates. Ceasing a SFP in the rural Ethiopia resulted with 7 % higher dropout rate for girls than the control group supporting the fact that SFP in Ethiopia results in better education outcomes^(46)^.

In many developing countries, primary school enrolment rates are high, but attendance is consistently low^(47)^. On top of poor health and short-term hunger, poor households fail to send children to school since they need them to work on farm or give care to siblings^(47)^.

A similar qualitative study in Ghana confirmed that teachers, caterers, parents and school administrators agreed with the fact that SFP had an impact on enrolment, attendance, completion and the academic performances of primary schoolchildren, in addition to cognitive development and the reduction of hunger^(48)^.

This study showed that the Addis Ababa SFP had an outstanding medium effect on educational performance of beneficiary students. Stunting and wasting were hindering conditions that determined educational performance. Thus, the longer the school meals provision the better will likely be long-term nutritional and educational outcomes among the beneficiaries.

The Addis Ababa SFP also had similar marginal medium effect among boys’ enrolment while its effect for girls’ enrolment was nil or too little to claim. An overall enrolment through time and mainly attraction of a higher number of boys each year is most likely associated with that significant number of boys in Addis Ababa go for search of income, child labour or other means than going to school as findings out of KII also support.

Potential reduction in absenteeism or consistent school attendance observed among schoolchildren without heterogeneity between sexes was a commendable effect of the Addis Ababa SFP. Better nutritional status and dropping absenteeism went parallel, reflecting the effect of the Addis Ababa SFP.

The SFP has helped girls to attentively attend class and attracted more boys, especially in the adolescent age, to schools while removing them out of child labour or street begging, and improved convenient environment for effective teaching–learning process.

The KIIs findings support this empirical evidence that teachers and school directors have noticed a potential improvement in absenteeism.

## Conclusion

The current findings highlighted notable effects of the Addis Ababa SFP on educational outcomes, specifically, on enrolment, academic performance and attendance, while reducing the prevalence of malnutrition. Thus, the programme would support to build the physical, mental and cognitive conditions of school-age children and adolescents as well as maintain their socio-economic wellbeing in later ages, of adulthood, as better health, nutrition and education contribute to productivity. This also implies that SFP could serve efforts toward the global sustainable development agenda as it can reach schoolchildren through human development-related interventions, including health, nutrition and education. Apart from alleviating hunger in schools and enhance nutritional outcomes, strengthening the program would inevitably help efforts to diminish educational inequalities.

## Supplementary material

For supplementary material accompanying this paper visit https://doi.org/10.1017/S1368980022000799. click here to view supplementary material
