# Supplementary material for: Impact of school meals on educational outcomes in Addis Ababa, Ethiopia
Source: Public Health Nutr. 2022 Mar 28;25(9):2614–24. doi: 10.1017/S1368980022000799 (PMC9991771; doi:10.1017/S1368980022000799)
Supplement: Supplementary file 1 [file S1368980022000799sup001.docx]

**Supplementary Table**

*Supplementary Table 1 Effect sizes of SFP on average scores of schoolchildren*

| Age | Sex | F | (df1, df2) | Sphericity assumption | | | Epsilon-Squared |
| --- | --- | --- | --- | --- | --- | --- | --- |
|  |  |  |  | Huynh-Feldt epsilon | Greenhouse- Geisser epsilon | Box's conservative epsilon |  |
| 5-9 | Girls | 26.39*** | (2, 1306) | 0.8785*** | 0.8765*** | 0.5*** | 0.04 |
|  | Boys | 18.21*** | (2, 1514) | 0.8608*** | 0.8592*** | 0.5*** | 0.02 |
| 10-14 | Girls | 31.29*** | (2, 2089) | 0.8052*** | 0.8042*** | 0.5*** | 0.03 |
|  | Boys | 27.07*** | (2, 2539) | 0.8156*** | 0.8148*** | 0.5*** | 0.02 |
| 15-19 | Girls | 3.91** | (2, 108) | 0.9097** | 0.8873** | 0.5* | 0.05 |
|  | Boys | 2.98* | (2, 106) | 0.7064* | 0.6961* | 0.5* | 0.04 |
| Overall | Girls | 62.22*** | (2, 3588) | 0.8382*** | 0.8375*** | 0.5*** | 0.03 |
|  | Boys | 48.44*** | (2, 4256) | 0.8315*** | 0.8309*** | 0.5*** | 0.02 |
|  | Both | 108.9*** | (2, 7846) | 0.8343*** | 0.834*** | 0.5*** | 0.03 |

***p<0.001, **p<0.05, *p<0.1

NB: Sphericity assumption tests are significant that the variances of the differences between all pairwise combinations of groups are equal.
